# The Effect of Cofactor Binding on the Conformational Plasticity of the Biological Receptors in Artificial Metalloenzymes: The Case Study of LmrR

**DOI:** 10.3389/fchem.2019.00211

**Published:** 2019-04-10

**Authors:** Lur Alonso-Cotchico, Jaime Rodríguez-Guerra Pedregal, Agustí Lledós, Jean-Didier Maréchal

**Affiliations:** ^1^Departament de Química, Universitat Autònoma de Barcelona, Barcelona, Spain; ^2^Stratingh Institute for Chemistry, University of Groningen, Groningen, Netherlands

**Keywords:** molecular modeling, artificial metalloenzymes, molecular dynamics, interactive analysis, cofactor binding, molecular plasticity

## Abstract

The design of Artificial Metalloenzymes (ArMs), which result from the incorporation of organometallic cofactors into biological structures, has grown steadily in the last two decades and important new-to-Nature reactions have been reached. These type of exercises could greatly benefit from an understanding of the structural impact that the inclusion of organometallic moieties may have on the biological host. To date though, our understanding of this phenomenon is highly partial. This lack of knowledge is one of the elements that condition that first-generation ArMs generally display relatively poor catalytic profiles. In this work, we approach this matter by assessing the dynamics and stability of a series of ArMs resulting from the inclusion, via different anchoring strategies, of a variety of organometallic cofactors into the Lactococcal multidrug resistance regulator (LmrR) protein. To this aim, we coupled standard force field-based techniques such as Protein-Ligand Docking and Molecular Dynamics simulations with a variety of trajectory convergence analyses, capable of assessing both the stability and flexibility of the different systems under study upon the binding of cofactors. Together with the experimental evidence obtained in other studies, we provide an overview on how these changes can affect the catalytic outcomes obtained from the different ArMs. Fundamentally, our results show that the convergence analysis used in this work can assess how the inclusion of synthetic metallic cofactors in proteins can condition different structural modulations of their host. Those conformational modifications are key to the success of the desired catalytic activity and their proper identification can be wisely used to improve the quality and the rate of success of the ArMs.

## Introduction

Incorporating homogenous catalysts into biological scaffolds (e.g., protein, DNA, or peptides) has become a common strategy to expand the scope of the biological space and produce biocompatible man-made biocatalysts (Schwizer et al., 2017; Diéguez et al., 2018). These *de novo* enzymes, also referred as Artificial Metalloenzymes (ArMs), can be generated under numerous strategies including post-translational approaches i.e., supramolecular (Ohashi et al., 2003; Mahammed and Gross, 2005; Reetz and Jiao, 2006; Bos et al., 2015), covalent (Reetz et al., 2002; Bos et al., 2013), or dative (Kokubo et al., 1983; Van De Velde et al., 2000) interactions, or eventually the incorporation of unnatural amino acids (UAA) (Drienovská et al., 2015, 2017) via sequencing approaches or by the direct expression through cellular vectors. The ArM design process can be divided in two different stages: (1) discovery, when a first catalytically efficient biohybrid shows some activity for a given reaction, and (2) optimization, when the initial candidates are chemically and/or genetically altered to reach improved activity in terms of yield, substrate selectivity, or regiospecificity. Whatever the stage at which the ArM design stands, the most important and complex molecular variable that needs to be controlled by designers is the stability of the interactions between the host and the artificial cofactor: a *sine qua non* condition which reaches pre-reactive resting states and catalytically competent geometries after the binding of the substrate.

Foreseeing the quality of the host-cofactor complementarity requires extensive molecular knowledge and remains a challenging exercise in the path of achieving experimentally efficient candidates. In fact, experimentalists base most of their design on trial-and-error strategies until they reach a first hit. In a way, designers are engaged in an unfair battle against evolution since they try to find good enough affinities between two moieties that never occur in Nature. One of the predominant variables for defining the quality of the interaction between the two entities, is the conformational adaptation of the receptor upon binding of the cofactor. In fact, for any *de novo* design, one of the major weaknesses is the poor consideration of protein dynamics along the designing exercises (Hammes-Schiffer and Benkovic, 2006; Henzler-Wildman and Kern, 2007; Nagel and Klinman, 2009; Hammes et al., 2011; Callender and Dyer, 2014; Maria-Solano et al., 2018). This is probably the reason why the catalytic efficiency of the new candidates is frequently many orders of a magnitude lower than that achieved by naturally-occurring enzymes (Jiang et al., 2008; Röthlisberger et al., 2008; Siegel et al., 2010). Despite the increasing number of ArMs over the last decades, little has been done to estimate the sensitivity of the biological host to the insertion of non-natural cofactors and how this, in turn, conditions the nature of the resting state of the ArM prior to any catalytic step. In this matter, *in silico* methods can be very helpful.

Molecular Modeling has been widely used to decode the nature of the dynamical events involved in structure-function relationship of naturally-occurring biological macromolecules. From short to large scale motions, theoretical (e.g., Molecular Dynamics (MD) simulations and Normal Modes Analysis) studies constantly provide evidence on how the dynamics of the protein host is influenced by the presence or absence of substrates and/or inhibitors as well as the tight relationship between these changes and the accessibility to catalytically efficient configurations (Dutta and Mishra, 2017; Sen et al., 2017; Sharma et al., 2017; Wilson and Wetmore, 2017; Wilson et al., 2017; Kamariah et al., 2018; Luirink et al., 2018; Rout et al., 2018; Schlee et al., 2018). It is therefore a legitimate question to use computation to assess to what extent bridging chemical and biological entities disturb the natural conformational space of the biomolecules and how damaging/beneficial this can be for catalysis.

Our group entered the field of ArM about a decade ago and focused both on understanding the mechanism of non-natural enzymes as well as providing protocols for the design of new systems. Our strategies are mostly based on integrated protocols, where physical models could range from Quantum Mechanics (QM) to Molecular Mechanics (MM) approaches (Muñoz Robles et al., 2015). One of the questions we wanted to solve is the magnitude of the conformational rearrangement experienced by the biological host under cofactor inclusion, a phenomenon that requires substantial computational improvements of MM methodologies in order to simulate metal-mediated recognition processes. In previous works we decoded the electronic origin of the control of the binding site motions and helix re-arrangements in the ArM constructed by the insertion of salophen into heme-oxygenase apo-enzyme (Muñoz Robles et al., 2011). Another interesting case came from describing how the inclusion of organometallic complexes into a protein scaffold can alter its structure leading to significant variations in the catalytic outcomes (Drienovská et al., 2017; Villarino et al., 2018). These studies provide us with some clues about the structural sensitivity of the receptor upon inclusion of the organometallic moiety, but no clear tendencies could be drawn as they were comprised of a case-specific analysis. To shed light on this matter we will perform a structural assessment benchmark, focusing on a unique receptor loaded with different homogeneous catalysts.

**Figure 1:**
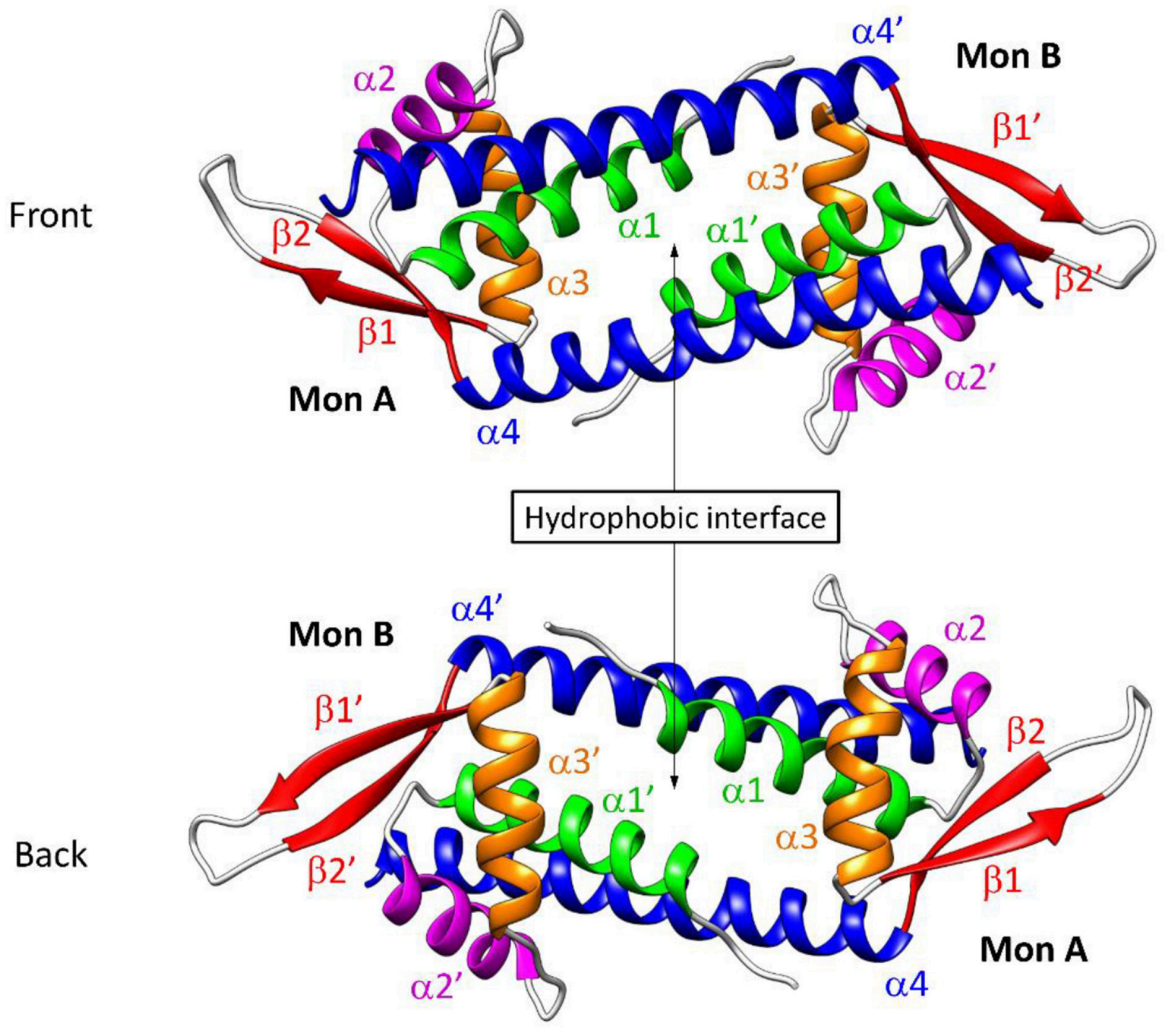
Representation of the reconstructed structure of the LmrR protein, labeled as described by Madoori et al. (2009) (PDB: 3F8F). The native form of the protein is constituted by two monomers which contain four α helices and one β-hairpin loop each.

Over the past few years, Roelfes et al. have focused on the Lactococcal multidrug resistance Regulator (LmrR) protein–a transcriptional repressor from the *Lactococcus lactis* organism–as a biological host for a variety of organometallic cofactors, leading to a set of enantioselective artificial metalloenzymes, including hydratases (Bos et al., 2013; Drienovská et al., 2015, 2017), cyclopropanases (Villarino et al., 2018) and Diels-Alderases (Bos et al., 2012). LmrR is a homodimeric protein with a particularly flat and hydrophobic dimer interface, capable of packing foreign aromatic molecules at the patch constituted by the tryptophan's W96/W96' of chains α4/α4', respectively, which are located at the center of the cavity (Figure 1).

**Figure 2:**
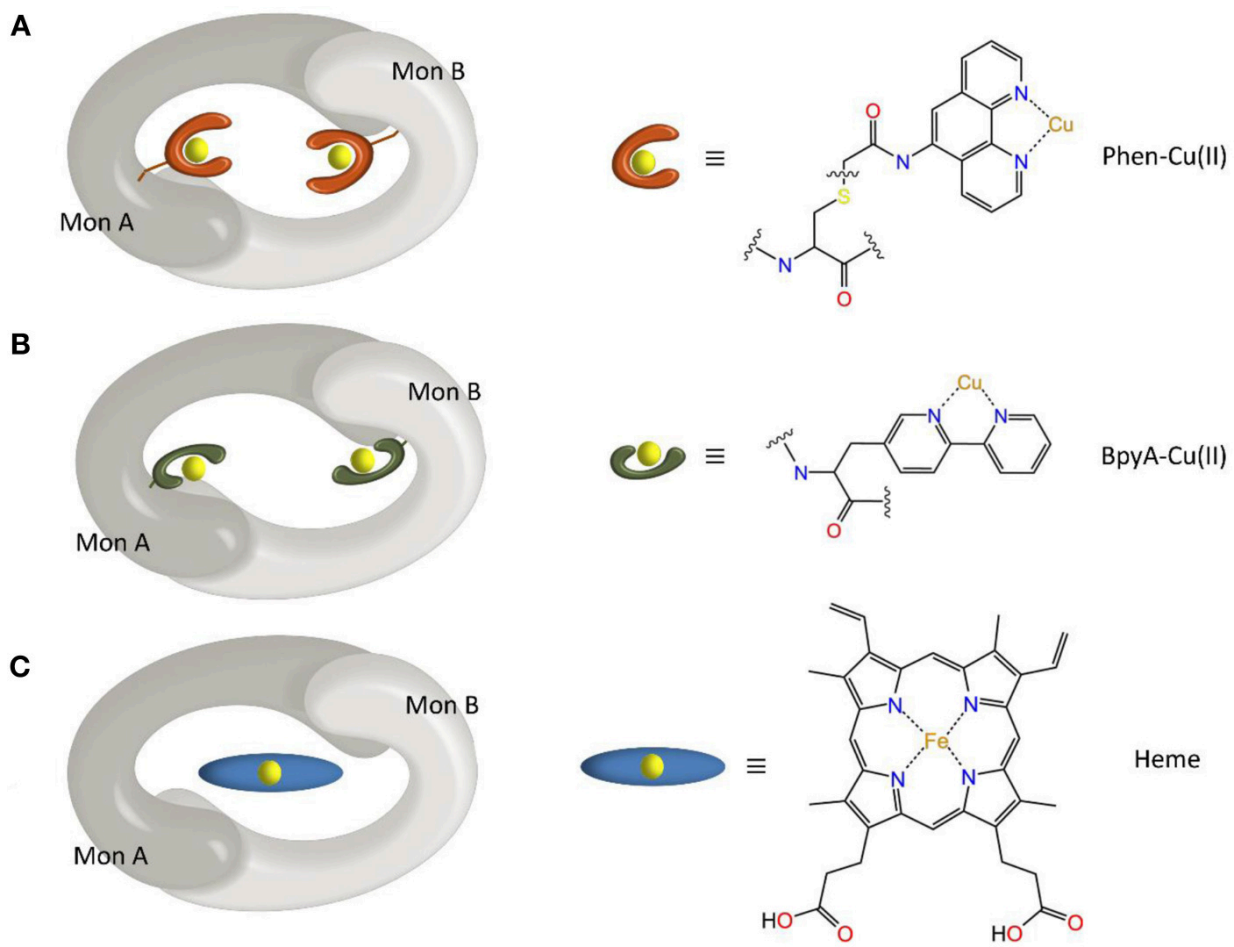
Scheme of the different ArMs considered in this work, constituted by the LmrR dimeric protein with linked **(A)** Phen-Cu(II) or **(B)** BpyA-Cu(II) cofactors to both monomers (Mon A and Mon B), or **(C)** an embedded heme group as designed by Roelfes et al. (Bos et al., 2013; Drienovská et al., 2015; Villarino et al., 2018).

Most of the new LmrR-based designs are the result of (a) the post-translational inclusion of a phenanthroline moiety (Figure 2A) or (b) the expression of the (2,2'-bipyridin-5yl)alanine (BpyA) unnatural amino acid (Figure 2B), at positions 89/89' of the dimeric LmrR protein. Both of them are nitrogen-based ligands of copper(II) ions and presented interesting activity for the hydration of ketones as well as the Friedel-Crafts alkylation reaction for either LmrR or DNA based catalysis (Arnold, 2009; Boersma et al., 2010; Bos et al., 2013; Drienovská et al., 2015, 2017). Separately, the same protein was used as a scaffold for the supramolecular recognition of hemin, resulting in an ArM with acquired cyclopropanase activity (Figure 2C) (Villarino et al., 2018). Recently, it has been employed to covalently attach a Rh(I) complex, forming a biohybrid capable of hydrogenating CO_2_ (Laureanti et al., 2019). Those works therefore present a unique opportunity to test how a given host could be sensitive to the insertion of different cofactors into their binding site.

From a computational point of view, dealing with bioorganometallic systems represents one of the most challenging modeling tasks, due to the necessity of describing biometallic interactions under standard force fields (an area still in strong development, Riccardi et al., 2018), while also accounting for possible wide structural variations resulting from embedding net charges (the metal center) in a natural hydrophobic environment. As a result, the identification of a set of *in silico* modeling techniques sensitive enough to predict structural variations that arise from the incorporation of inorganic moieties into the protein host, is fundamental to speed up the success rate of ArM designs.

**Figure 3:**
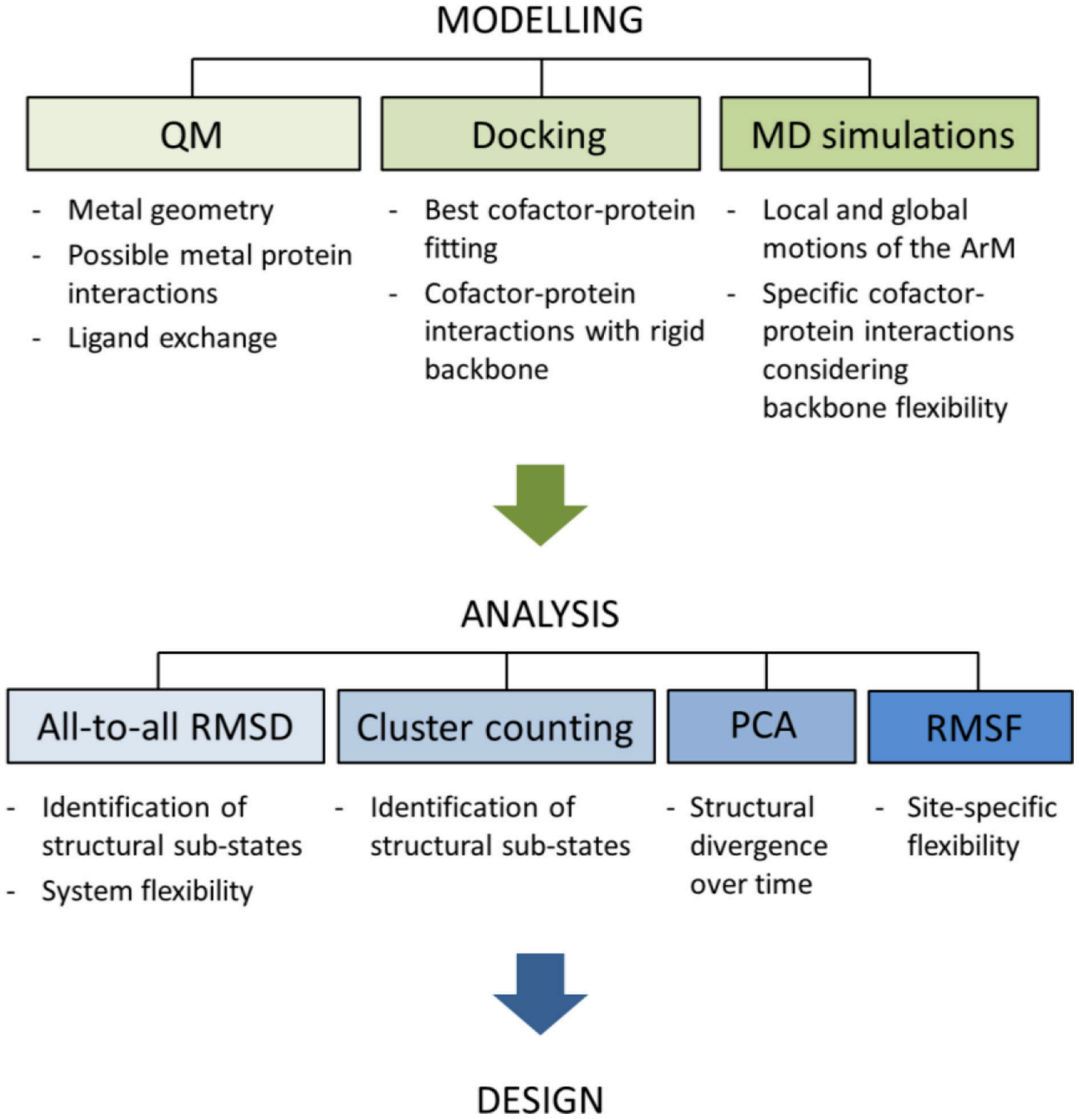
Schematic representation of the workflow applied for all the systems discussed in the present article. Notice that this work is focused on the stage of analysis and, thus, the modeling part is not discussed. For further details about the modeling process see the Supplementary Material.

Here, we focus on the dynamical implications of inserting non-natural organometallic cofactors in the core of the LmrR protein. The computational approach consists of the combination of Protein-Ligand Docking and MD simulations followed by diverse trajectory analyses including all-to-all Root-mean-square-deviation (RMSD), Principal Component Analysis (PCA), cluster counting, and Root-mean-square-fluctuation (RMSF) approaches (see Figure 3). Together with the visual inspection of the trajectories, these analyses provide valuable information on two main areas: (1) the MD simulation time-scale required to ensure a proper conformational exploration by the hybrid systems, and (2) how the inclusion of different organometallic external moieties can promote conformational variations of the same biological host.

## Materials and Methods

### Construction of the Dataset

Together with two natural forms of the LmrR protein found in the Protein Data Bank (PDB), the models include: the *apo* form of the LmrR protein (1), the LmrR protein bound to its inhibitor daunomycin (2), the ArMs resulting from the supramolecular interaction between the heme group and LmrR (3) and the linking of the biaqua form of two Phen-Cu(II) (4) or BpyA-Cu(II) (5) cofactors to the positions M89C/M89C' of the LmrR protein, as illustrated in Table 1.

**Table 1:** Definition of the different systems considered in this work.

**System**	**Description**	**Binding**	**Model**	**Bound molecule**
1	*apo* LmrR	–	3F8B	–
2	LmrR + daunomycin	Supramolecular	3F8F	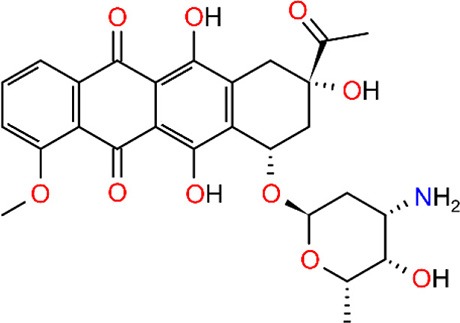
				Daunomycin
3	LmrR + heme	Supramolecular	6FUU	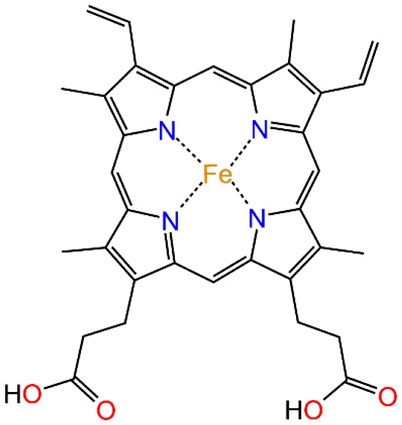
				Heme
4	LmrR + 2Phen-Cu(II)	Covalent (post-translational)	Docking	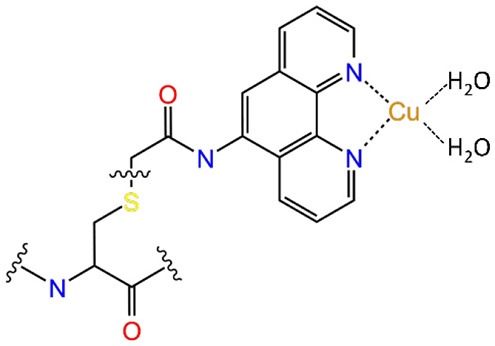
				Phen-Cu(II)-(H_2_O)_2_
5	LmrR + 2BpyA-Cu(II)	Covalent (UAA)	Docking	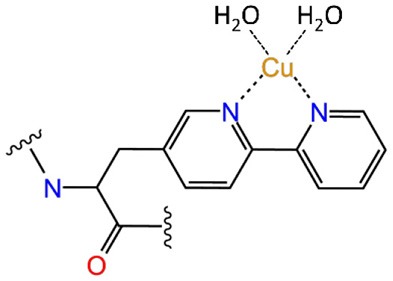
				BpyA-Cu(II)-(H_2_O)_2_

Initially, quantum calculations were performed to obtain the optimized structures of the different organometallic cofactors. All the complexes were optimized with Gaussian 09 (Frisch et al., 2009) at the DFT level using the B3LYP-D3 (Becke, 1993; Stephens et al., 1994; Grimme et al., 2010) functional. For all the non-metallic atoms the 6-31G(d,p) basis set was used. For the iron and copper atoms the SDD effective core potential and its associated basis set (Dolg et al., 1987) including *f* functions for (Ehlers et al., 1993) was employed. For systems 4 and 5, for which no X-ray data was available, the organometallic complexes were incorporated into the LmrR protein scaffold via a covalent Protein-Ligand Docking approach, considering flexible side-chains for all the residues pointing toward the active site. For systems 1, 2, and 3 the X-ray structures of the *apo* LmrR (PDB code: 3F8B), the LmrR bound to daunomycin drug (PDB code: 3F8F), or the LmrR bound to the heme group (PDB code: 6FUU), respectively, were used as a starting point for the MD simulations. For system 3, the crystallographic data shows several orientations of the heme group corresponding with a rotated porphyrin with respect to the perpendicular axis passing across the iron ion. Thus, in this case, the selection of the starting point was based on a consensus between the X-ray and docked structures, resulting in the orientation corresponding to the best scored pose. Daunomycin (for system 1) and crystallographic water molecules were manually removed. Systems 4 and 5 were constructed via a covalent Protein-Ligand Docking approach by linking the aqua bound form of both BpyA-Cu(II) and Phen-Cu(II) cofactors, respectively, to the position 89/89' of the LmrR, using the X-ray structure bound to its inhibitor daunomycin (PDB code: 3F8F) as a scaffold. All docking runs were performed with GOLD 5.2 (Verdonk et al., 2003). The best scored structures, together with the X-ray data for systems 1, 2 and 3, were embedded in boxes of around 37,000 water molecules and were used as the starting point for 300 ns MD simulations. The simulations were run with the OpenMM 7.0 engine (Eastman and Pande, 2010) as wrapped in the OMMProtocol program (Pedregal et al., 2018).

The resulting trajectories were processed with an in house-automated procedure that computes a series of complementary analysis to assess the convergence: (1) the changes in the RMSD of the coordinates with respect to the starting structure, (2) the RMSD evolution with respect to all the frames in the simulation, (3) a PCA on the structural variability of the backbone (Balsera et al., 1996; David and Jacobs, 2014), and (4) a cluster counting method (Daura et al., 1999; Smith et al., 2002), as described in the following section. Root-mean square fluctuation (RMSF) plots were also calculated for all systems to identify the regions with a wider range of movement in each trajectory. For depiction purposes, the dihedral angles of each trajectory were analyzed with time-structure Independent Components Analysis (tICA) (Naritomi and Fuchigami, 2013) and clustered by k-means with MSMBuilder (Harrigan et al., 2017). The resulting structures were superposed with UCSF Chimera's *matchmaker* command.

### A Few Words About the Analysis of MD Trajectories

The most common strategy to analyze trajectories for assessing the structural stability of biological macromolecules is based on aligning the structure along the simulation and computing the root mean square deviation (RMSD) against a reference structure, which helps to determine whether the simulation has stabilized around a given average conformation. While easy to understand and calculate, RMSD analyses fail to show the nature of the conformational states that are sampled and only provide partial information about the structural steadiness of the system. As a result, several authors have proposed additional procedures to obtain deeper information about the convergence status of MD simulations (Daura et al., 1999; Smith et al., 2002; Grossfield and Zuckerman, 2009; Knapp et al., 2011). One of them is the all-to-all RMSD, which, instead of calculating the RMSD against a single structure, considers all the possible conformations along the simulation, resulting in a two-dimensional matrix of RMSD measurements. The resulting plot helps to depict the different visited regions along the MD trajectory, which might be challenging to detect with a standard RMSD analysis. Another noteworthy analytical strategy is the cluster counting tool which, in consensus with the above method, is useful to identify the rate of the appearance of new sub-states along the MD trajectory. Last, the Principal Component Analysis (PCA) is a standard statistical procedure used to study the correlation within a dataset (Abdi and Williams, 2010). Concerning the scope of this work, it allows the visual inspection of the conformational space explored along the simulation timescale and whether the system continues to visit new regions or not, which enables the user to see at a glance how stable the system is and therefore whether the trajectory is approaching convergence or not. As recommended by Grossfield and Zuckerman (2009), the integration of these four complementary analytical tools can provide an accurate assessment of the conformational space explored as well as the degree of convergence of a MD trajectory.

To compute the four analyses of the trajectories, we set up several scripts using Python 3.6 through the Jupyter Notebook interface (Kluyver et al., 2016). CPPTraj (Roe and Cheatham, 2013) was first used to remove the waters and reimage the system within the same periodic box. MDTraj (McGibbon et al., 2015) was then used to load and align the trajectories. This library also provided the RMSD calculations. PCA was calculated with the routines available in scikit-learn (Pedregosa et al., 2011) set to generate two components. Cluster counting followed the algorithm proposed in Smith et al. (2002), with a RMSD cut-off of 2.0 Å. Figures were plotted with matplotlib 2.0 (Hunter, 2007). In all cases, only the α-carbons belonging to the α-helix segments were considered for distance calculations, thus discarding highly flexible regions of the protein scaffold as evidenced in the per-residue root mean square fluctuation (RMSF) plots (Figure S6 RMSF).

The methods described above are able to depict the magnitude of the changes along a MD simulation but not what the nature of these changes are; i.e., which regions of the protein are involved in those structural variations. For this reason, their use in consensus with both the visual inspection of the trajectories as well as tools able to identify local and global motions is of great importance. In this regard, the structures obtained from tICA and k-means clustering can be very helpful. Since tICA tends to group the structure changes explained by slower motions in the first components (Naritomi and Fuchigami, 2013), it can be used to distinguish between rapid and slow motions as the system evolves over time, since these are normally related to local and global structural changes of the system, respectively. This allows the identification of the structural features that lead to the variability/stability detected by the above methods and, thus, a clearer assessment of the structural impact promoted by the incorporation of the different external moieties into the protein scaffold.

For further details about the computational procedure the reader is referred to the Supplementary Material.

#### Non-metallic Cofactor Bound LmrR

To provide a reference to assess the impact of the incorporation of artificial metallic cofactors in the LmrR protein scaffold, we first studied two experimental structures of LmrR available at the PDB without any metallic moieties bound: an *apo* form of the LmrR (PDB code: 3F8B) (Table 1, system 1) and a LmrR form bound to the daunomycin inhibitor (PDB code: 3F8F) (Table 1, system 2). This inhibitor is a substantially large and hydrophobic molecule and consequently quite reminiscent of the cofactors that have been studied in this work.

The 300 ns-long Molecular Dynamics simulations of the *apo* (system 1) and daunomycin (system 2) form of LmrR revealed local conformational changes that mainly involve the ends of helixes α4 and α4' and the β hairpin loops of both monomers (Figure S1). For system 1, in addition, collective motions, not found in system 2, related to the opening and closing of the LmrR interdimeric binding site were elucidated as illustrated in Figures S1, S2. It appears that the presence of the daunomycin inhibitor, which is sandwiched between tryptophan's W96/W96' of helix α4/α4' at the center of the cavity, does not significantly influence the global motility of the protein scaffold in contrast to the *apo* form of LmrR (Figures S1, S3, systems 1 and 2). The lack of the hydrophobic inhibitor at the dimer interface seems to promote the closing of the pore by bringing the helices α4/α4' closer: around 8 Å between the alpha carbons of tryptophan's W96/W96', which are located in the center of helix α4/α4' (from now this parameter will be used as reference to assess the opening/closing of the dimer interface; see Figure S2, system 1). In contrast, the drug-bound form maintains an opened arrangement of helices α4/α4' (around 12.5 Å; see Figure S2, system 2).

The combination of cluster counting, all-to-all RMSD and PCA analyses (Figures S3–S5, systems 1 and 2) provides more data about the conformational sampling for systems 1 and 2. The appearance of new structural clusters reaches its plateau at 150 ns of the MD trajectory for both systems, which suggests a converged structural sampling for the simulation time scale. This is consistent with the all-to-all RMSD analysis, which indicates that both systems fluctuate between well-defined sub-states, which is also associated with a converged trajectory (Figure S4, systems 1 and 2). These sub-states are related to the structures which present certain structural divergences with respect to the X-ray structure, consistent with the local/global motions described above, associated mostly to the flexibility of the β hairpin loops and the ends of the α4/α4' helixes and, only for system 1, the closing of the dimer interface (Figure S5, systems 1 and 2).

Altogether, these analyses show that the natural motions of LmrR involve mostly the interdominial interface between monomers A and B, as well as their relative rotation, which is smoothed in the inhibitor-bound form of the protein. Additionally, this state is associated with a wide active cavity resulting from direct hydrophobic interactions of W96/W96', A92/A92', and V15/V15' with the daunomycin inhibitor. From these results we can conclude, on one hand, that a time scale of 150 ns is enough to reach a proper conformational sampling in the MD simulations for the *apo* and the daunomycin-bound forms of LmrR and, on the other hand, that, overall, the accommodation of hydrophobic moieties at the hydrophobic interface would reduce the global conformational plasticity of LmrR.

#### The Heme-Based Artificial Metalloenzyme

**Figure 4:**
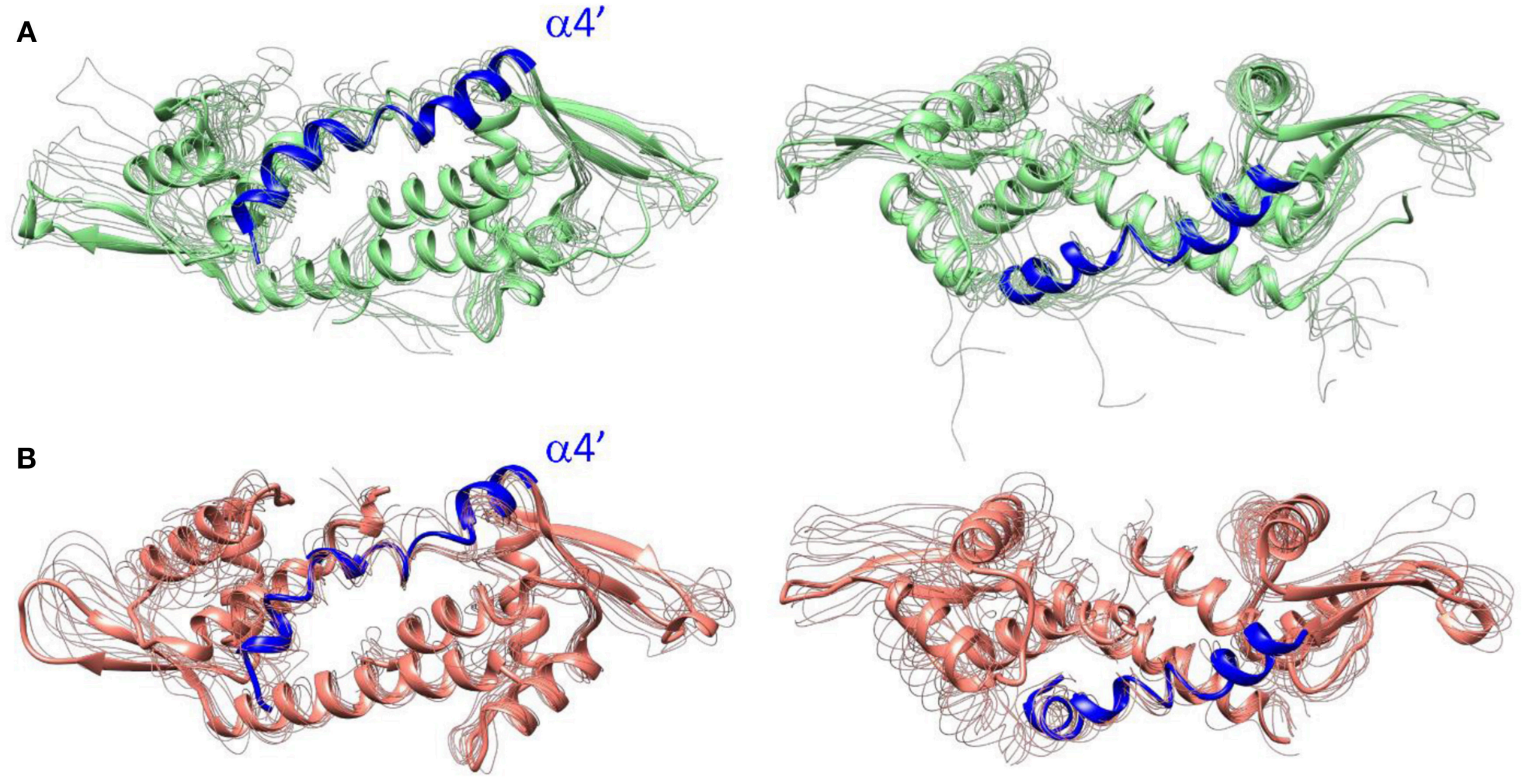
Front (left) and top (right) views of LmrR corresponding with frames extracted from 300 ns of MD simulation. They are comprised of a selection of the representative structures among the most divergent k-means clusters extracted from tICA for **(A)** LmrR including the drug daunomycin (system 2) and **(B)** LmrR bound to the heme group (system 3).

Our study then focused on the Artificial Metalloenzyme systems. We started with the ArM resulting from the supramolecular interaction between the heme group and the LmrR protein. Recent studies showed that the LmrR-heme system is able to reach efficient cyclopropanation profiles (Villarino et al., 2018). The X-ray structure of the LmrR bound to heme (PDB: 6FUU) displays the prosthetic group sandwiched in between the two α4/α4' helices of the homodimers with major hydrophobic interactions between the macrocycle and the side chains of the two tryptophan's W96/W96', as well as polar interactions between N19/N19' and N14/14' and the carboxylate groups of the heme moiety. Due to the similar size and configuration between the heme group and the inhibitor daunomycin in the LmrR active site, we expected a similar impact on the protein dynamics between systems 2 (daunomycin-bound) and 3 (heme-bound). Interestingly, results showed that the dynamics of both systems followed the same trend: in both cases, the presence of the planar hydrophobic molecule at the dimer interface promotes an increase in the flexibility of the α4' helix, which for the LmrR⊂heme ArM occurs with more plasticity, comprising mainly the distortion of the same helix (Figure 4). This effect promotes the closing of the hydrophobic interface with respect to system 2 (Figure S2, systems 2 and 3), which is broader than for system 1 but narrower than for system 2, and is reflected in the increased amount of sub-states found for this system: cluster counting shows that, in this case, MD simulation reaches convergence with a maximum of 12 clusters of structures, in contrast to systems 1 and 2, which reach convergence in a maximum of eight clusters of structures (Figure S3). In addition, all-to-all RMSD and PCA analyses show not only a higher amount of sub-states related with a major structural exploration of the system (dark zones covering the diagonal of the all-to-all RMSD plot), but also a greater flexibility (light areas in the background of the all-to-all RMSD plot), and divergence on the nature of these sub-states (no overlapping spots in the PCA plot).

These results show that the presence of an organometallic complex at the hydrophobic interface does not change the dynamical tendency of the natural form of LmrR in system 2 but promotes an increase on the plasticity of the system, that could only be deciphered by the use of cluster counting and PCA analysis, and was particularly related to the motion of the helix α4'. More likely, this effect can be explained by the presence of the porphyrin metal center, absent in the daunomyicin-bound system 2, at the LmrR active site. Interestingly, the observed motion of the helix α4' was associated with the catalytic activity of the LmrR⊂heme ArM, in which the displacement of the tryptophan W96', located in this helix, was key for vacating one of the axial faces on the porphyrin and, thus, making the metal center accessible to the substrates to reach pre-catalytic states for the cyclopropanase activity (Villarino et al., 2018).

#### The Copper-Based Artificial Metalloenzymes

Next, we wanted to assess the effect of incorporating organometallic cofactors but, in this case, covalently linked to the protein scaffold at positions 89/89'. These were the copper-bound nitrogenated compounds, Ala-bipyridine (BpyA) and phenanthroline (Phen), which are covalently linked to the protein scaffold at positions 89/89'. Interestingly, despite sharing same chemical properties and a similar size and overall planarity, previous reports have shown greatly different catalytic outcomes for the enantioselective addition of water to conjugated ketones (Bos et al., 2013; Drienovská et al., 2017). These differences have been associated to the different lengths of the linkers that bind the aromatic rings of the nitrogenated moieties with the backbone (Table 1, systems 4 and 5), which seems to affect the overall behavior of the artificial systems (Drienovská et al., 2017). Thus, we decided to include these ArMs in our study to identify the key elements that promote such a differential effect.

For that purpose, we studied the conformational variability of the LmrR scaffold loaded with Phen-Cu(II) and BpyA-Cu(II) cofactors simulating their most likely biaqua resting state (Table 1, systems 4 and 5). These were included into both monomers of the LmrR protein scaffold via a covalent Protein-Ligand Docking procedure, showing a good affinity with the protein binding site (Table S1) in both cases, being slightly better for Phen-Cu(II)-(H_2_O)_2_ (38.89 ChemScore units) than for BpyA-Cu(II)-(H_2_O)_2_ (34.85 ChemScore units). Consistently with previous systems, the best score poses were selected and used as the starting point for 300 ns of Molecular Dynamics simulation.

**Figure 5:**
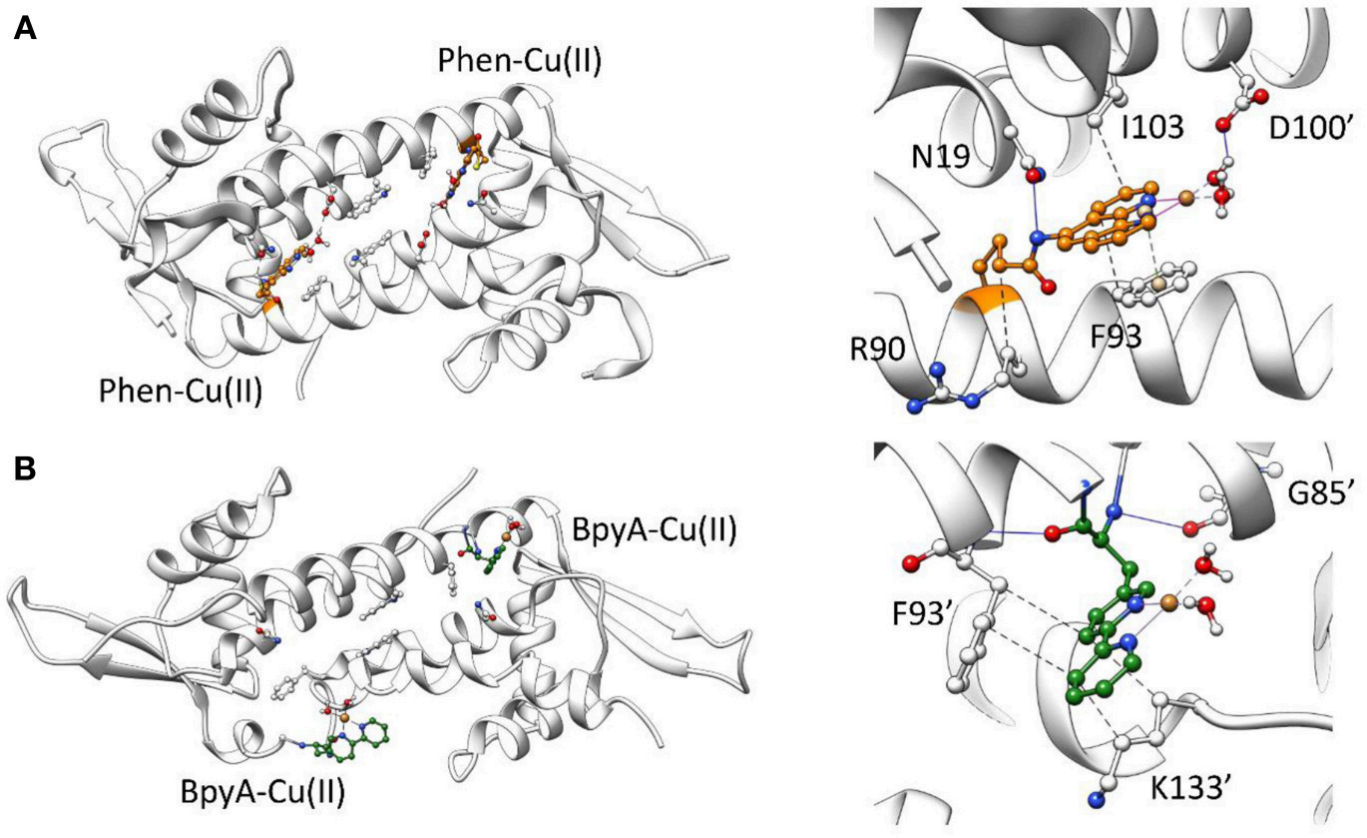
Representative structures along 300 ns of MD simulation for the LmrR protein containing **(A)** the Phen-Cu(II)-(H_2_O)_2_ cofactor and **(B)** the BpyA-Cu(II)-(H_2_O)_2_ cofactor. A general view of the system (left) as well as specific interactions between the copper cofactors and surrounding residues analyzed with Plip (Salentin et al., 2015) (right) is illustrated.

Due to their structural similarities, it would be expected that the inclusion of the Phen and BpyA cofactors into LmrR promotes a similar impact on the scaffold than the heme organometallic cofactor (system 3). However, both systems 4 and 5 present strongly different dynamic tendencies, which are particularly noteworthy for the Phen-containing ArM. Results showed a strong decrease of the global motions and the plasticity of the system with respect to the systems 1, 2, and 3 (Figures S4–S6, systems 1–4), the main motions being reduced to the ends of the α4/α4' helices. The appearance of new clusters of sub-states converges at the very beginning of the simulation, without identification of changes along the MD time-scale, which is consistent with the very low plasticity of the system as shown in Figure S3 for system 4 (only 3 clusters of structures were identified after 300 ns of MD simulation). In this configuration, the biaqua form of the Phen-Cu(II) cofactors appeared stabilized by interactions comprising mainly π-stacking with phenylalanine residues F93/F93' and hydrophobic contacts with I103/I103' and the side chains of the R90/R90' residues. In addition, hydrogen bonds between the tail of the cofactor with N19/N19' residues as well as between the waters bound to the metal center and the aspartates D100/D100' seemed to further stabilize the location of the cofactor at the entrance of the dimer interface (Figure 5A). This binding mode is accompanied by a broader dimer interface (around 14 Å), resulting, for the daunomycin bound system 2, from the hydrophobic interactions between the active site residues with the aromatic cofactor. Additionally, all-to-all RMSD and PCA analysis evidenced a significantly reduced plasticity of the LmrR⊂Phen system (Figures S3, S4, system 4). The former analysis showed few sub-states, especially well-defined and with strong presence after 50 ns of MD simulation (the big dark area which center lies at the diagonal of the plot). Furthermore, the later analysis showed that between the few sub-states identified there is a very low divergence (PCA data appears superimposed). These results evidence that the LmrR system loaded with the Phen-Cu(II) cofactors presents major stability than that of the natural form of LmrR (system 1). It is of great relevance to note that, in contrast to the flexible nature of the catalytically active Lmr⊂heme system (system 3), this ArM, which presents the highest stability among all those considered in this study, showed a very good catalytic activity, in this case for the enantioselective hydration of ketones (Bos et al., 2013). This observation suggests that one of the factors driving the promiscuity of the LmrR based artificial enzymes is also related to the flexibility of the protein backbone, which needs to be controlled in order to perform the different catalytic transformations.

**Figure 6:**
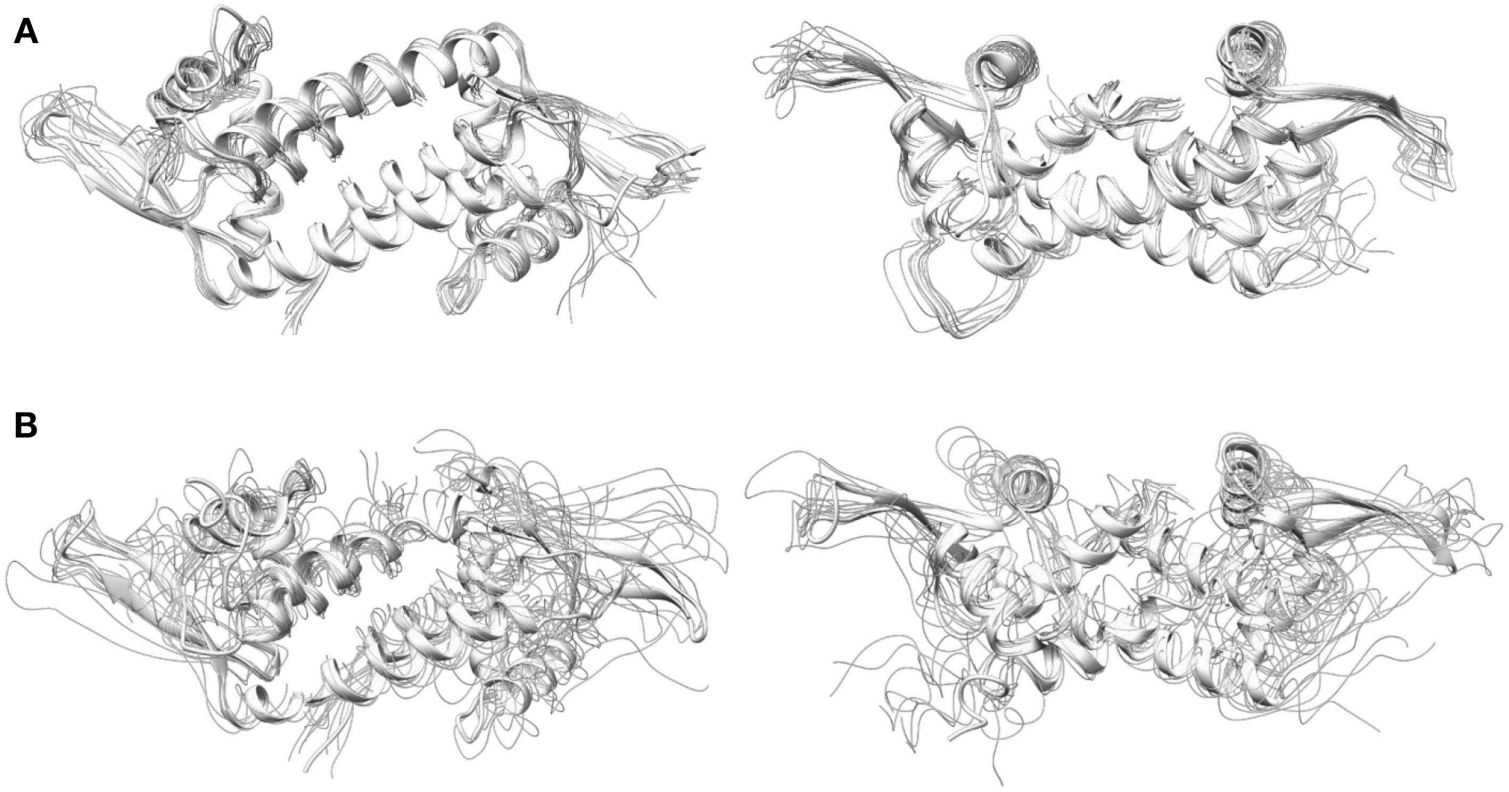
Front (left) and top (right) view of LmrR corresponding with frames extracted from 300 ns of MD simulation comprising the 10 most representative structures extracted from k-means clustering in the tICA space for **(A)** LmrR bound to the Phen-Cu(II)-(H_2_O)_2_ cofactor (system 4) and **(B)** LmrR bound to the BpyA-Cu(II)-(H_2_O)_2_ cofactor (system 5).

Regarding system 5, the results showed a totally different scenario. The MD simulation evidenced the poor capability of the BpyA-Cu(II)-(H_2_O)_2_ cofactors to reach the center of the cavity. Instead, they appeared pointing toward the solvent during most of the trajectory and at any time along 300 ns of MD simulation the complexes appeared embedded at the dimer interface. This is the result of a lack of hydrophobic and polar interactions between the active site residues and the bipyridine cofactors (Figure 5B). The bipyridine cofactor has a shorter tail linking the bipyridine rings and the protein backbone (the beta carbon) than phenanthroline (five atoms, see Figure 2), which makes the complexes lie out of the hydrophobic cavity (Figure 5B). Consequently, this lack of aromatic ligands at the dimer interface promotes a narrower arrangement between helixes α4/α4' (around 8 Å between the alpha carbons of W96/W96' residues, see Figure S2 system 5), the same as the distance observed for the *apo* form of LmrR (system 1). Additionally, the lack of stability in the positioning of the bipyridine cofactors is extended to the rest of the protein backbone, promoting an increase in its plasticity as well as distorting the global protein structure (Figure 6). This behavior is well-captured by the cluster counting, all-to-all RMSD and PCA analysis (Figures S3–S5). As expected, the number of identified sub-states highly increased for this system in contrast to system 4 (LmrR⊂Phen), being grouped in a total of nine clusters of structures along 300 ns of MD simulation, in contrast to the three clusters identified for system 4. In addition, they showed a more flexible system (light areas in the background of the all-to-all RMSD plot) as well as a strong divergence of the identified sub-states (the spots in the PCA plot do not superimpose along 300 ns of MD simulation). RMSF analysis also revealed this difference: system 5 showed higher average fluctuation values for all chains (see Figure S6). Interestingly, this ArM was not able to efficiently perform the enantioselective hydration of ketones, presenting much lower levels of both conversion and enantioselectivity in contrast to the LmrR⊂Phen ArM (Bos et al., 2013; Drienovská et al., 2017). After deciphering the mechanism of system 5 (Drienovská et al., 2017), it was revealed that an aspartate located at helix α4' was responsible for boosting the nucleophilic attack, the first step of the hydration reaction. For this to occur in an enantioselective manner, the cofactor-substrate complex needed to be not only located inside the active site but also stabilized in only one orientation. For this reason, the stability of the interactions at the active site seems key for the desired reaction to proceed in the LmrR-based artificial hydratases (systems 4 and 5), in contrast to the LmrR⊂heme ArM, which requires the flexibility of helix α4' to reach pre-catalytic structures.

In summary, the results showed that the stabilizing interactions that occur between the active site residues and the copper cofactors seem crucial in providing stability to the global motions of the LmrR scaffold. In this regard, the hydrophobic and polar interactions and the length of the cofactor linkers play a critical role in reducing or increasing the flexibility of the system. Additionally, it is reasonable to think that the presence of non-stabilized metal moieties close to the protein backbone is one of the factors that may promote the strong distortion of the protein backbone as observed for the LmrR⊂BpyA (system 4). As a result, only the LmrR⊂Phen ArM is able to maintain an asymmetric environment around the copper cofactor during the entire simulation time scale, which is reflected in the catalytic efficiency of this artificial hydratase (Bos et al., 2013).

## Conclusions

This study aims to shed light on questions concerning the structure-function relationship in proteins, especially focusing on organometallic-containing systems such as ArMs. Dealing with organometallic moieties adds an extra layer of difficulty to modeling and simulation exercises due to the challenging task of describing non-natural metal-containing moieties as part of the standard force fields, as well as their structural effect when interacting with the biological scaffold. Thus, the identification of a computational modeling workflow, together with an analytical protocol sensitive enough to decipher the changes that result from the incorporation of external moieties into proteins, appears to be of great relevance, especially in the enzyme design field.

To guarantee the quality of the systems used in this study, we made use of a set of well-characterized ArMs (LmrR loaded with heme, copper-bound phenanthroline, or copper-bound bipyridine) designed by Roelfes et al. whose models have been experimentally validated in previous works (Drienovská et al., 2017; Villarino et al., 2018) to perform a comparative analysis of simulations resulting from the combination of Quantum Mechanics, Protein-Ligand Docking, and Molecular Dynamics techniques. Our results provide evidence that the convergence analysis used in this work can help explain the structural trends of the different systems under study. They show how the insertion of different non-natural metallic cofactors into the same biological scaffold may condition different structural modulations that, in addition, are key to the success of the desired catalytic activity. Put into the context of ArM design and *in silico* exercises, it is therefore crucial to first assess (or, at least, consider this magnitude as a variable to control sooner or later in the designing pipeline) the degree of rigidity/flexibility of the receptor-cofactor partner throughout MD simulations to understand how this can affect the reaction mechanism of interest.

Aiming at including dynamical notions in computer-aided design of ArMs, here we show the strength of combining an integrative strategy (docking + MD simulations) with convergence-based analysis, including all-to-all RMSD, PCA, RMSF, and cluster counting, to characterize the structural behavior of these complex organometallic systems. Our results also contribute to the debate on the benefit of accounting for stable vs. flexible protein scaffolds to drive the designs of the first generations of ArMs. This work makes clear that, due to the high amount of degrees of freedom controlling the different catalytic mechanisms occurring in ArMs, each of them must be considered as a separate system with its own particular patterns and features (like flexibility/rigidity): ideally, their specific structural requirements would need to be evaluated on a one-by-one basis. Unfortunately, this means that we are still far from establishing a universal metric to guide the design of any ArM. Thus, at present, we find it essential, at least, to account for a proper protocol that establishes which modeling and analytical tools, such as the ones selected for this work, will ensure the gain of enough structural knowledge before investing in further efforts.

## Author Contributions

JR-G and LA-C performed the calculations and analysis. J-DM designed the project. JR-G, LA-C, and J-DM discussed the analysis and wrote the manuscript. AL participated in the discussion and the writing of the manuscript.

### Conflict of Interest Statement

The authors declare that the research was conducted in the absence of any commercial or financial relationships that could be construed as a potential conflict of interest.
