# Supplementary material for: The Effect of Cofactor Binding on the Conformational Plasticity of the Biological Receptors in Artificial Metalloenzymes: The Case Study of LmrR
Source: Front Chem. 2019 Apr 10;7:211. doi: 10.3389/fchem.2019.00211 (PMC6467942; doi:10.3389/fchem.2019.00211)
Supplement: Supplementary file 1 [file Data_Sheet_1.docx]

# The Effect of Cofactor Binding on the Conformational Plasticity of the Biological Receptors in Artificial Metalloenzymes: The Case Study of LmrR.

**Supplementary information**

Lur Alonso-Cotchico,^1,2^ Jaime Rodríguez-Guerra Pedregal,^1^ Agustí Lledós,^1^ Jean-Didier Maréchal^1^*

^1^Departament de Química, Universitat Autònoma de Barcelona, Edifici C.n., 08193 Cerdanyola del Vallés, Barcelona, Spain

^2^Stratingh Institute for Chemistry, University of Groningen, Nijenborgh 4, 9747 AG Groningen, the Netherlands

*email: JeanDidier.Marechal@uab.cat

**Model systems**

Available X-ray data of the LmrR was used for constructing the *apo* LmrR (PDB code: 3F8B), the drug-bound (PDB code: 3F8F) and the heme-bound form (PDB: 6FUU) of LmrR. For the ArMs containing both the Phen-Cu(II) and BpyA-Cu(II) based complexes corresponding to systems **4** and **5**, for which no X-ray data was available, the scaffold containing drug daunomycin (system **2**) was used. The inhibitor was manually removed and then the cofactors (Phen-Cu(II)-(H_2_O)_2_ and BpyA-Cu(II)-(H_2_O)_2_) were included at position 89 of LmrR. For all the models the crystallographic water molecules and ions were manually removed and missing amino acids in the X-ray material were reconstructed by superimposition of the LmrR monomers with UCSF Chimera’s matchmaker command (Pettersen et al. 2004).

**Computational methods**

*Quantum calculations.* All the organometallic complexes Phen-Cu(II)-(H_2_O)_2_, BpyA-Cu(II)-(H_2_O)_2_ and the heme group were optimized with Gaussian09 program (Frisch et al. 2009) at DFT/B3LYP-D3 level (Becke 1993; Stephens et al. 1994; Grimme et al. 2010) and the 6-31g(d,p) basis set (Petersson et al. 1988; Petersson and Al-Laham 1991; Rassolov et al. 2001). The implicit Solvation Model based on Density (SMD) (Marenich, Cramer, and Truhlar 2009) was used as continuum method. For non-metallic atoms the SDD pseudopotential (Dolg et al. 1987) including a *f* polarization function for the outer electrons (the inner core electrons were described by the SDD effective core potential (ECP)) was used.

*Protein-Ligand Docking*. The optimized structures of the complexes Phen-Cu(II)-(H_2_O)_2_ and BpyA-Cu(II)-(H_2_O)_2_ were docked at position 89/89’ of LmrR. For this purpose, M89 and M89’ were first mutated to cysteine or to alanine, using the Dunbrack rotamer library (Dunbrack Jr 2002) available in the UCSF Chimera program (Pettersen et al. 2004), to construct systems **4** and **5**, respectively. A covalent approach was used by superimposing the Cα (alanine) or the S atom (cysteine) with the corresponding atom in the organometallic complexes. All docking runs were carried out with GOLD 5.2 program (Verdonk et al. 2003) and results were evaluated using the ChemScore (Eldridge et al. 1997) scoring function.

*Classical Molecular Dynamics simulations*. The X-ray data as well as the best scored docking solutions for systems **4** and **5** were used as starting point for 300 ns of MD simulation. The parameters for all the organometallic complexes, including the heme, Phen-Cu(II)-(H_2_O)_2_ and BpyA-Cu(II)-(H_2_O)_2_ were generated based on standard approaches: point charges were calculated with antechamber (Wang et al. 2006) according to the RESP procedure (Bayly et al. 1993). Bonded terms involving the metal center were calculated based on the Seminario’s approach (Seminario 1996). The remaining atoms were parameterized according to GAFF force field (Wang et al. 2004). Lacking terminal residues were replaced by uncharged motifs suggested in the AMBER force field (ACE and NME) (Cornell et al. 1995).

The MD was set up with the xleap program (Cornell et al. 1995). The systems were immersed into a cubic box containing about 37,000 water molecules, considering 10 Å from the protein to the edge of the box, and 4 chloride counter ions to neutralize the simulation cell. The TIP3P (Jorgensen et al. 1983) and Amber ff99SB-ILDN (Cornell et al. 1995) force fields were used to describe water and the protein, respectively. Chloride ions were described based on the ions94.lib library (Cornell et al. 1995).

The Particle Mesh Ewald method (Darden, York, and Pedersen 1993) was used for the calculation of long-range electrostatic interactions, and a cut off of 10 Å was stablished for Van der Waals and short-range electrostatics interactions. The SHAKE algorithm (Ryckaert, Ciccotti, and Berendsen 1977) was used for constraining bonds involving hydrogen atoms. The equation of motion was integrated with a Langevin integrator (Schneider and Stoll 1978; Brünger, Brooks III, and Karplus 1984) and a time step of 1 fs. A Monte Carlo barostat set at 1.01325 bar (Duane et al. 1987) to achieve constant temperature and pressure.

The simulation was run with OpenMM 7.0 program (Eastman and Pande 2010) following an standard procedure automatized with OMMprotocol (Pedregal et al. 2018). The system was first energy minimized during 3000 steps to allow progressively water molecules, side-chain and backbone to move. Then, the temperature was increased from 100 K up to 300 K for thermalization of water molecules and side chains. Last, 300 ns of production under NPT conditions was carried out.

Trajectory analysis was carried out with Python 3.6.6 and a combination of MDTraj v1.9.1 (McGibbon et al. 2015), PyTraj (as included in AmberTools 18) (Nguyen et al. 2017, 2016), MSMBuilder v3.8 (Harrigan et al. 2017), scikit-learn v0.18.2 (Pedregosa et al. 2011), SciPy v1.1 (Jones et al. 2007) and NumPy v1.12.1 (Van Der Walt, Colbert, and Varoquaux 2011). Plots were drawn with Matplotlib v3.0.0 (Hunter 2007) and MSMExplorer v1.1.0 (Cronkite-Ratcliff and Pande 2013). All code is provided in separate files meant to be executed in the Jupyter Notebook environment (v5.7) (Ragan-Kelley et al. 2014). Graphical illustrations were created with UCSF Chimera program (Pettersen et al. 2004).

**Tables and figures**

**Table S1**. Best poses from docking of complexes A) Phen-Cu(II)-(H_2_O)_2_ and B) BpyA-Cu(II)-(H_2_O)_2_ into the LmrR scaffold.

| Ligand | mon1 | mon2 | ChemScore | ΔG | S_hbond_ | S_metal_ | S_lipo_ | H_rot_ | ΔE_clash_ | ΔE_int_ | ΔE_cov_ | intcor | S_protein_ |
| --- | --- | --- | --- | --- | --- | --- | --- | --- | --- | --- | --- | --- | --- |
| Phen-Cu(II) | A | - | 38.89 | -26.67 | 0.00 | 0.00 | 207.23 | 1.19 | -13.21 | 0.58 | 0.41 | 1.21 | 0.00 |
|  | A | A | 38.96 | -26.63 | 0.00 | 0.00 | 206.88 | 1.19 | -12.96 | 0.36 | 0.27 | 1.21 | 0.00 |
| BpyA-Cu(II) | B | - | 34.85 | -15.96 | 0.00 | 0.00 | 89.54 | 0.00 | -19.07 | 0.00 | 0.17 | 0.00 | 0.00 |
|  | B | B | 34.34 | -16.24 | 0.00 | 0.00 | 91.93 | 0.00 | -18.37 | 0.00 | 0.26 | 0.00 | 0.00 |


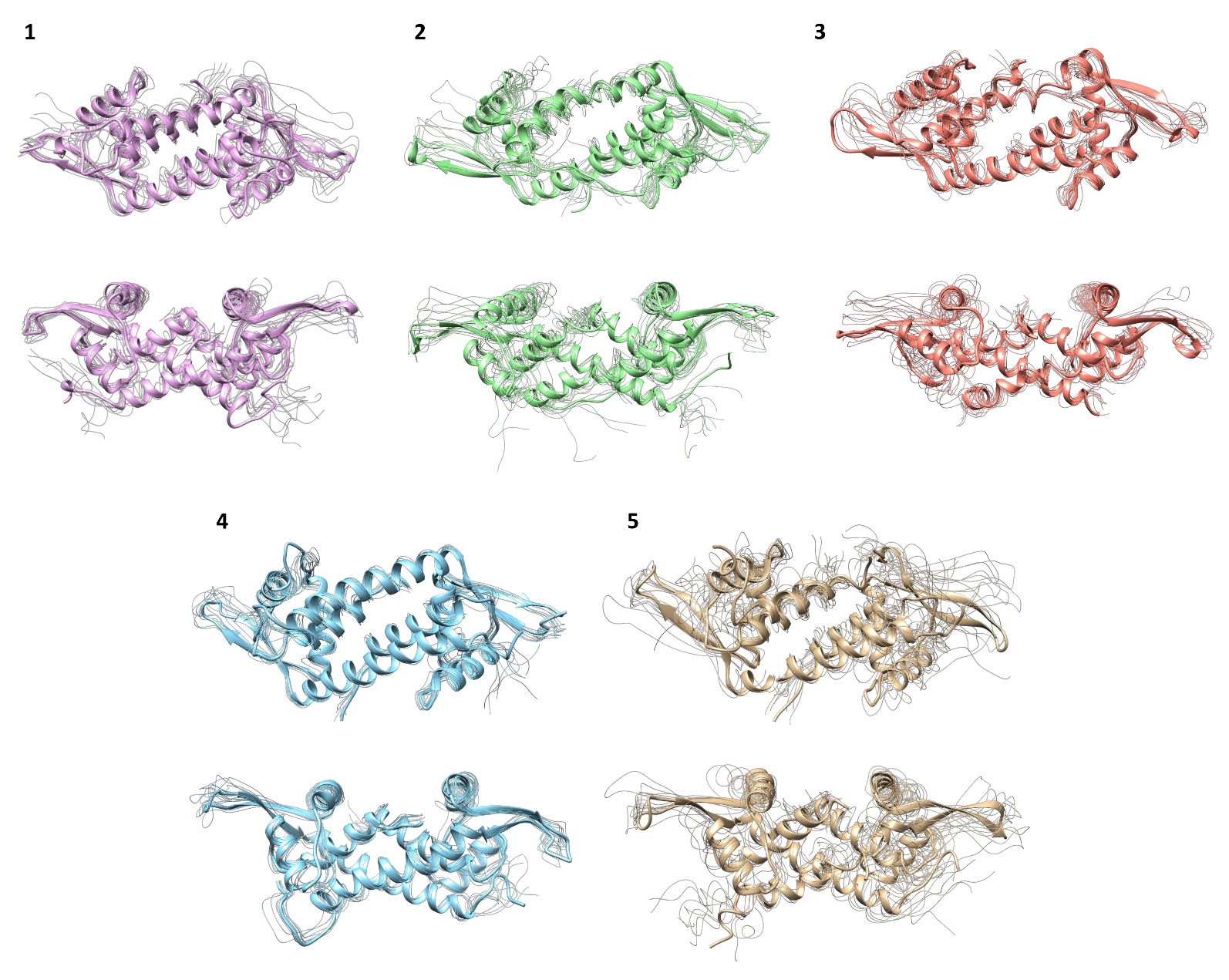


**Figure S1**. Front and rear views of the representative structures extracted from k-means clusters in the tICA space found along 300 ns of MD simulation for the different LmrR-based systems under study: the apo form (**1**), the drug-bound form (**2**), the heme-based Arm (**3**) and the copper cofactors Phen-Cu(II) (**4**) and BpyA-Cu(II) (**5**) based ArMs.

**
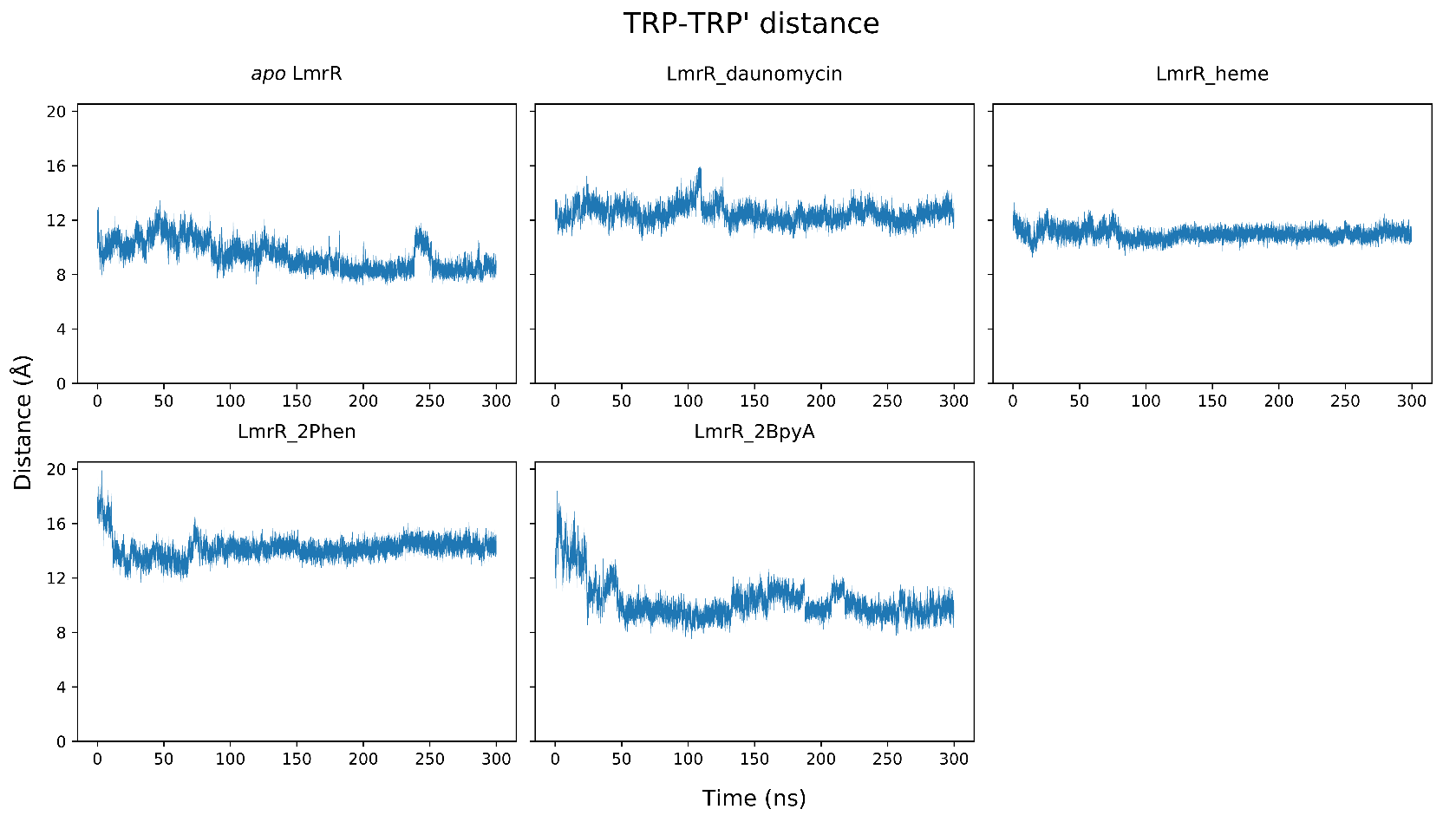
**

**Figure S2.** Distance between helices α4/α4’ measured along the 300 ns of MD simulation, as represented by the α carbon atoms of tryptophan residues at positions 96 and 96’.


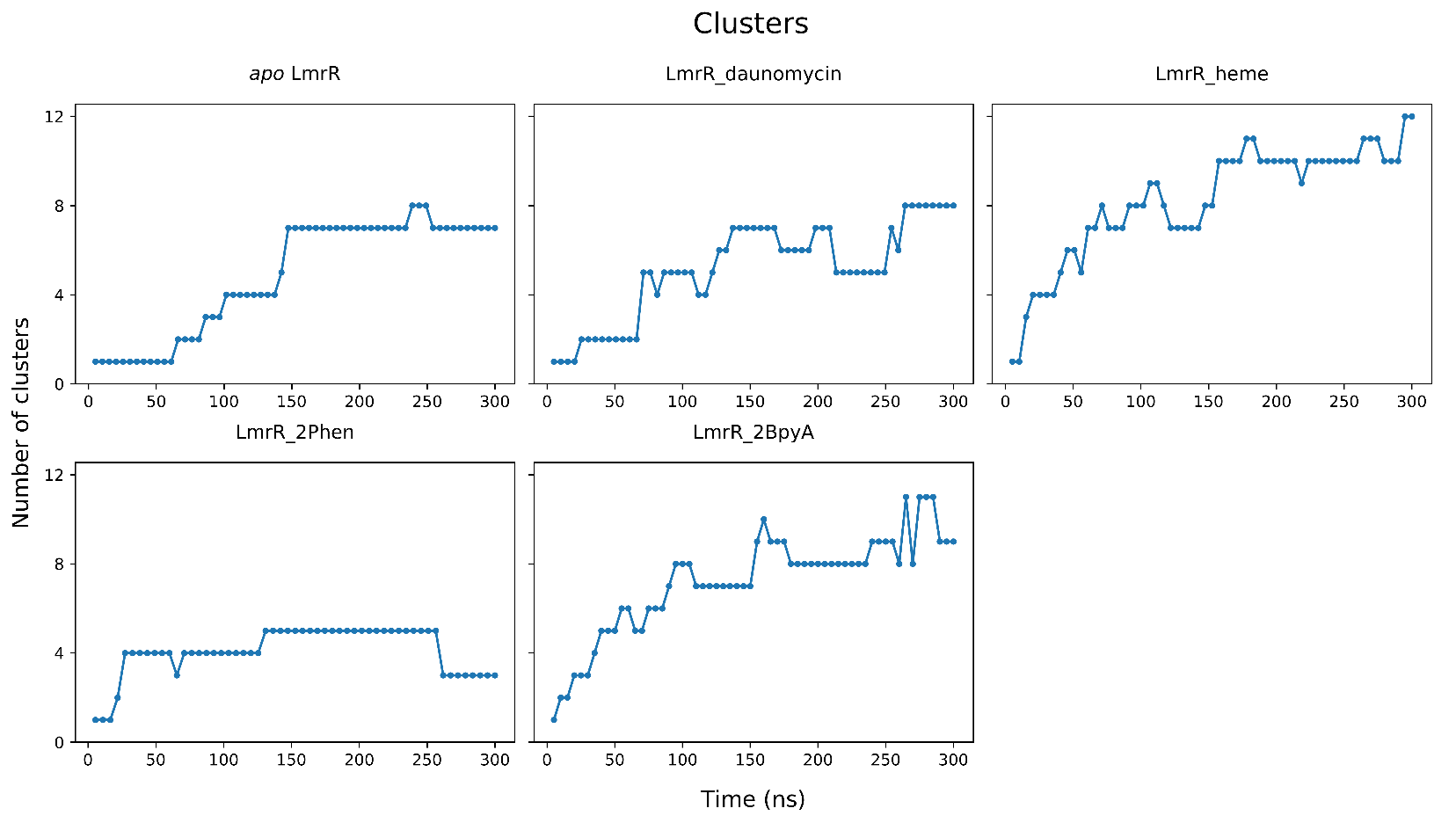


**Figure S3**. Cluster counting analysis from 300 ns of MD simulation for the LmrR-based systems under study (left to right, top to bottom): the apo form (**1**), the drug-bound form (**2**), the heme-based Arm (**3**) and the copper cofactors Phen-Cu(II) (**4**) and BpyA-Cu(II) (**5**) based ArMs. Clustering approach is based on grouping similar structures. As new regions of the conformational space are visited along the simulation time-scale, new clusters are obtained. A stable number of clusters (lack of new sub-states) is indicative of a converged trajectory, since the system should have visited all the conformational wells. In contrast, if the cluster counting plot keeps growing, the simulation can be considered no converged.


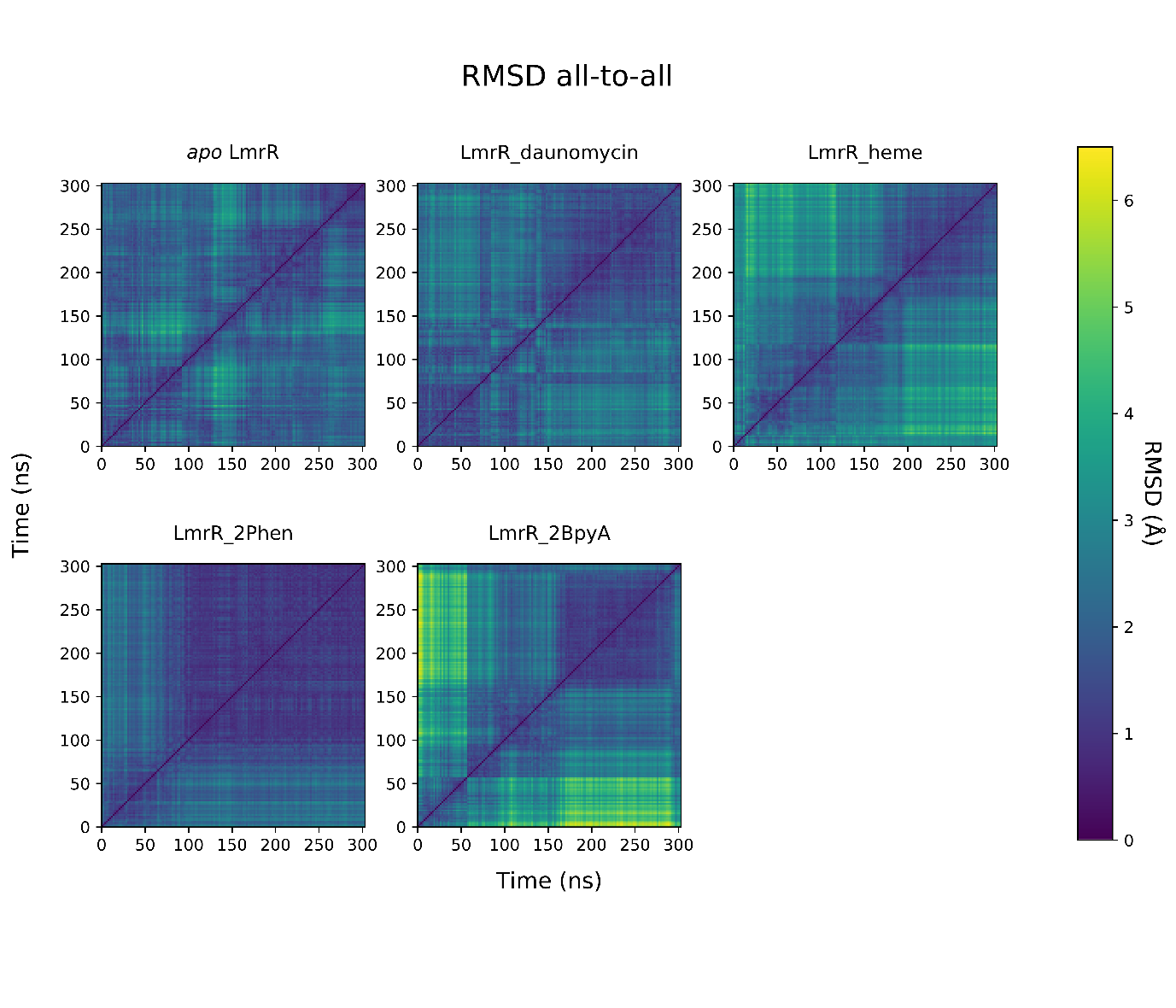


**Figure S4.** All-to-all RMSD analysis extracted from 300 ns of MD simulation for the LmrR-based systems under study (left to right, top to bottom): the apo form (**1**), the drug-bound form (**2**), the heme-based Arm (**3**) and the copper cofactors Phen-Cu(II) (**4**) and BpyA-Cu(II) (**5**) based ArMs. The color of the values plotted in the illustrated 2D figures indicates the RMSD values but resulting in a visually appealing and intuitive image. If only one main conformation is identified, this figure shows a relatively homogeneous color whose maximum value is within the established threshold (as for system **4**). When more sub-states are identified, the transitions are identified by clearly separate, colored patches according to the RMSD threshold. However, if a mix of small patches is observed along the diagonal, with no clear separation, then it will mean that the simulation is still visiting new regions of the conformational landscape.


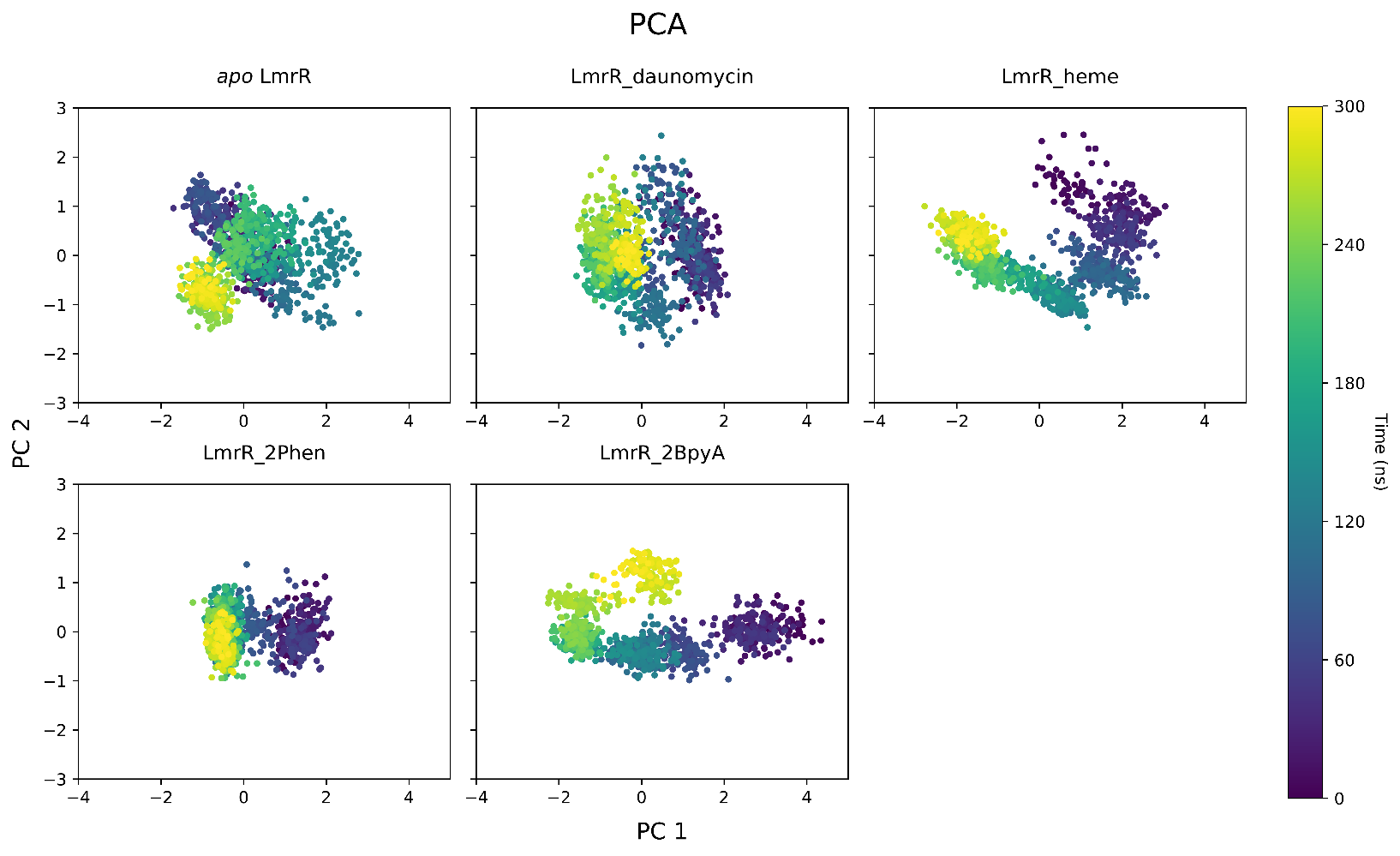


**Figure S5.** PCA analysis retrieved from 300 ns of MD simulation for the LmrR-based systems under study (left to right, top to bottom): the apo form (**1**), the drug-bound form (**2**), the heme-based Arm (**3**) and the copper cofactors Phen-Cu(II) (**4**) and BpyA-Cu(II) (**5**) based ArMs. In the resulting plot the color indicates the simulation time. When trajectories have converged, the PCA plot should show a concentration of points where late conformations often overlap or are close to early ones (high correlation), meaning that the same states are re-visited. In contrast, if the PCA plot features high dispersion of the data, then it can be assumed that the trajectory is still visiting new spaces.

**
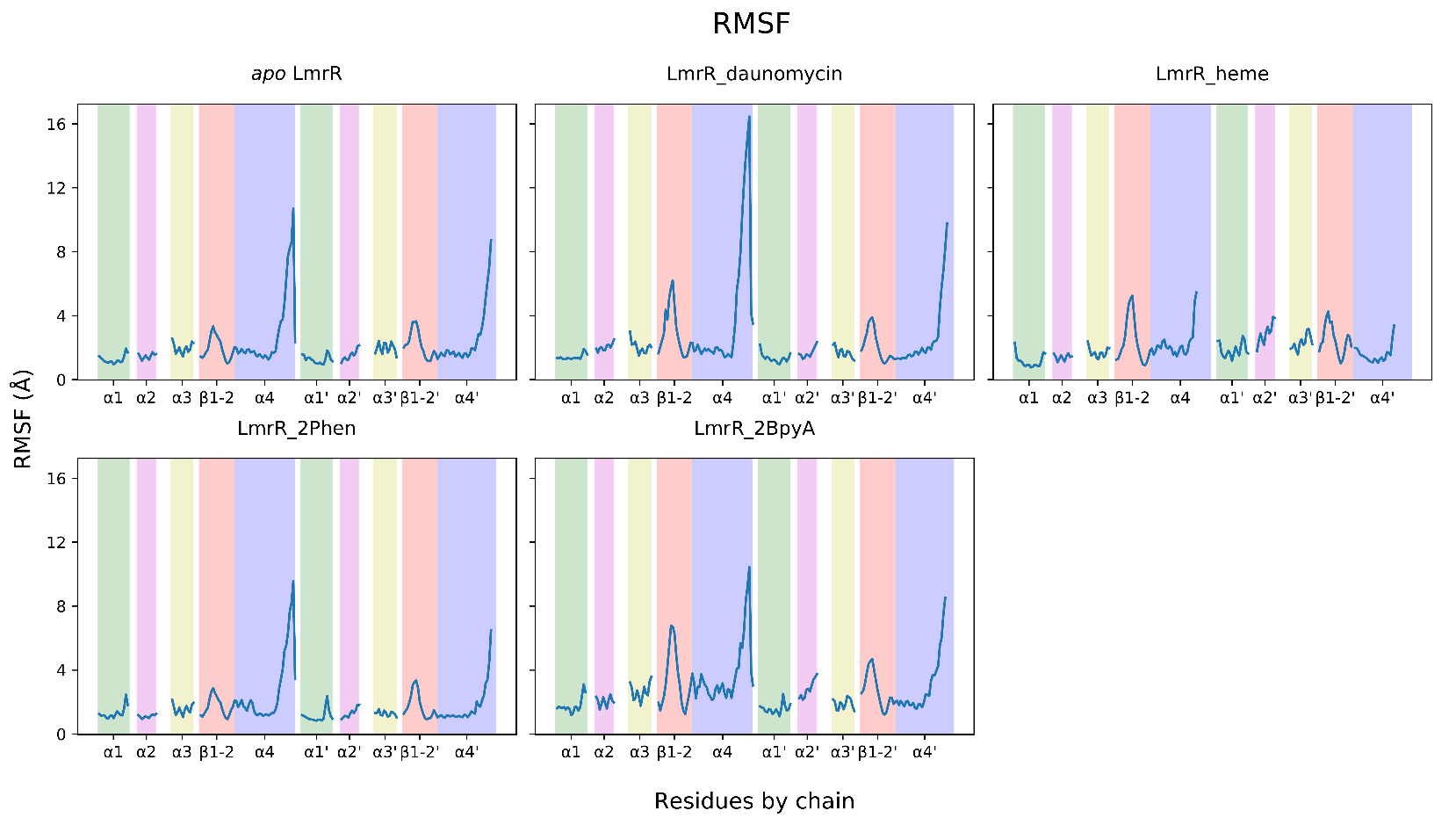
**

**Figure S6.** RMSF from 300 ns of MD simulation for the LmrR-based systems under study (left to right, top to bottom): the apo form (**1**), the drug-bound form (**2**), the heme-based Arm (**3**) and the copper cofactors Phen-Cu(II) (**4**) and BpyA-Cu(II) (**5**) based ArMs. The higher peaks correspond with the most flexible regions of the protein averaged long the simulation time-scale. The green strands are associated to the α1/α1’ helix, pink to α2/α2’, yellow to α3/α3’, red to β hairpin loops for monomers A and B, and blue to helices α4/α4’

**References**

Bayly, C.I., Cieplak, P., Cornell, W.D., and Kollman, P.A. (1993). A well-behaved electrostatic potential based method using charge restraints for deriving atomic charges: The RESP model. *J. Phys. Chem.* 97, 10269–80.

Becke, A.D. (1993). Density-functional thermochemistry. III. The role of exact exchange. *J. Chem. Phys.* 98, 5648–52.

Brünger, A., Brooks III, C.L., and Karplus, M. (1984). Stochastic boundary conditions for molecular dynamics simulations of ST2 water. *Chem. Phys. Lett.* 105, 495–500.

Cornell, W.D., Cieplak, P., Bayly, C.I., Gould, I.R., Merz, K.M., Ferguson, D.M., Spellmeyer, D.C., Fox, T., Caldwell, J.W., and Kollman, P.A. (1995). A second generation force field for the simulation of proteins, nucleic acids, and organic molecules. *J. Am. Chem. Soc.* 117, 5179–97.

Cronkite-Ratcliff, B., and Pande, V. (2013). MSMExplorer: visualizing Markov state models for biomolecule folding simulations. *Bioinformatics* 29, 950–52.

Darden, T., York, D., and Pedersen, L. (1993). Particle Mesh Ewald: an N·log(N) method for ewald sums in large systems. *J. Chem. Phys.* 98, 10089.

Dolg, M., Wedig, U., Stoll, H., and Preuss, H. (1987). Energy-adjusted ab initio pseudopotentials for the first row transition elements. *J. Chem. Phys.* 86, 866–72.

Duane, S., Kennedy, A.D., Pendleton, B.J., and Roweth, D. (1987). Hybrid monte carlo. *Phys. Lett. B* 195, 216–22.

Dunbrack Jr, R.L. (2002). Rotamer libraries in the 21st century. *Curr. Opin. Struct. Biol.* 12, 431–40.

Eastman, P., and Pande, V. (2010). OpenMM: a hardware-independent framework for molecular simulations. *Comput. Sci. Eng.* 12, 34–39.

Eldridge, M.D., Murray, C.W., Auton, T.R., Paolini, G. V, and Mee, R.P. (1997). Empirical scoring functions: I. The development of a fast empirical scoring function to estimate the binding affinity of ligands in receptor complexes. *J. Comput. Aided. Mol. Des.* 11, 425–45.

Frisch, M.J., Trucks, G.W., Schlegel, H.B., Scuseria, G.E., Robb, M.A., Cheeseman, J.R., Scalmani, G., and Barone, V. (2009). Gaussian 09, Revision D.01. *Gaussian 09, Revis. D.01*.

Grimme, S., Antony, J., Ehrlich, S., and Krieg, H. (2010). A consistent and accurate ab initio parametrization of density functional dispersion correction (DFT-D) for the 94 elements H-Pu. *J. Chem. Phys.* 132, 154104.

Harrigan, M.P., Sultan, M.M., Hernández, C.X., Husic, B.E., Eastman, P., Schwantes, C.R., Beauchamp, K.A., McGibbon, R.T., and Pande, V.S. (2017). MSMBuilder: statistical models for biomolecular dynamics. *Biophys. J.* 112, 10–15.

Hunter, J.D. (2007). Matplotlib: A 2D graphics environment. *Comput. Sci. Eng.* 9, 90–95.

Jones, E., Oliphant, T., Peterson, P., and others. (2007). SciPy: open source scientific tools for python. *Comput. Sci. Eng.*

Jorgensen, W.L., Chandrasekhar, J., Madura, J.D., Impey, R.W., and Klein, M.L. (1983). Comparison of simple potential functions for simulating liquid water. *J. Chem. Phys.* 79, 926–35.

Marenich, A. V, Cramer, C.J., and Truhlar, D.G. (2009). Universal solvation model based on solute electron density and on a continuum model of the solvent defined by the bulk dielectric constant and atomic surface tensions. *J. Phys. Chem. B* 113, 6378–96.

McGibbon, R.T., Beauchamp, K.A., Harrigan, M.P., Klein, C., Swails, J.M., Hernández, C.X., Schwantes, C.R., Wang, L.P., Lane, T.J., and Pande, V.S. (2015). MDTraj: a modern open library for the analysis of molecular dynamics trajectories. *Biophys. J.* 109, 1528–32.

Nguyen, H., Roe, D.R., Swails, J., and Case, D.A. (2016). PYTRAJ v1.0.0.dev1: Interactive data analysis for molecular dynamics simulations, January.

Nguyen, H., Swails, J., Roe, D.R., and Cody. (2017). Amber-MD/pytraj: v2.0.0, March.

Pedregal, J.R., Alonso-cotchico, L., Velasco-carneros, L., and Mare, J. (2018). OMMProtocol: a command line application to launch Molecular Dynamics simulations with OpenMM. In *ChemRxiv*.

Pedregosa, F., Varoquaux, G., Gramfort, A., Michel, V., Thirion, B., Grisel, O., Blondel, M., et al. (2011). Scikit-learn: Machine Learning in Python. *J. Mach. Learn. Res.* 12, 2825–30.

Petersson, G.A., and Al-Laham, M.A. (1991). A complete basis set model chemistry. II. Open-shell systems and the total energies of the first-row atoms. *J. Chem. Phys.* 94, 6081–90.

Petersson, G.A., Bennett, A., Tensfeldt, T.G., Al-Laham, M.A., Shirley, W.A., and Mantzaris, J. (1988). A complete basis set model chemistry. I. The total energies of closed-shell atoms and hydrides of the first-row elements. *J. Chem. Phys.* 89, 2193–2218.

Pettersen, E.F., Goddard, T.D., Huang, C.C., Couch, G.S., Greenblatt, D.M., Meng, E.C., and Ferrin, T.E. (2004). UCSF Chimera - A visualization system for exploratory research and analysis. *J. Comput. Chem.* 25, 1605–12.

Ragan-Kelley, M., Perez, F., Granger, B., Kluyver, T., Ivanov, P., Frederic, J., and Bussonnier, M. (2014). The Jupyter/IPython architecture: a unified view of computational research, from interactive exploration to communication and publication. *Am. Geophys. Union, Fall Meet. 2014, Abstr. Id. H44D-07*. http://adsabs.harvard.edu/abs/2014AGUFM.H44D..07R.

Rassolov, V.A., Ratner, M.A., Pople, J.A., Redfern, P.C., and Curtiss, L.A. (2001). 6-31G* basis set for third-row atoms. *J. Comput. Chem.* 22, 976–84.

Ryckaert, J.-P., Ciccotti, G., and Berendsen, H.J.C. (1977). Numerical integration of the cartesian equations of motion of a system with constraints: molecular dynamics of n-alkanes. *J. Comput. Phys.* 23, 327–41.

Schneider, T., and Stoll, E. (1978). Molecular-dynamics study of a three-dimensional one-component model for distortive phase transitions. *Phys. Rev. B* 17, 1302.

Seminario, J.M. (1996). Calculation of intramolecular force fields from second-derivative tensors. *Int. J. Quantum Chem.* 60, 1271–77.

Stephens, P.J., Devlin, F.J., Chabalowski, C.F., and Frisch, M.J. (1994). Ab initio calculation of vibrational absorption and circular dichroism spectra using density functional force fields. *J. Phys. Chem.* 98, 11623–27.

Verdonk, M.L., Cole, J.C., Hartshorn, M.J., Murray, C.W., and Taylor, R.D. (2003). Improved protein-ligand docking using GOLD. *Proteins* 52, 609–23.

Walt, S. Van Der, Colbert, S.C., and Varoquaux, G. (2011). The NumPy array: a structure for efficient numerical computation. *Comput. Sci. Eng.* 13, 22–30.

Wang, J., Wang, W., Kollman, P.A., and Case, D.A. (2006). Automatic atom type and bond type perception in molecular mechanical calculations. *J. Mol. Graph. Model.* 25, 247–60.

Wang, J., Wolf, R.M., Caldwell, J.W., Kollman, P.A., and Case, D.A. (2004). Development and testing of a general Amber force field. *J. Comput. Chem.* 25, 1157–74.
